# The Virological, Immunological, and Imaging Approaches for COVID-19 Diagnosis and Research

**DOI:** 10.1177/2472630320950248

**Published:** 2020-08-18

**Authors:** An Sen Tan, Sanjna Nilesh Nerurkar, Wei Chang Colin Tan, Denise Goh, Chi Peng Timothy Lai, Joe Poh Sheng Yeong

**Affiliations:** 1 Lee Kong Chian School of Medicine, Nanyang Technological University, Singapore, Singapore; 2 Yong Loo Lin School of Medicine, National University of Singapore, Singapore, Singapore; 3 Institute of Molecular Cell Biology (IMCB), Agency of Science, Technology and Research (A*STAR), Singapore, Singapore; 4 Department of Anatomical Pathology, Singapore General Hospital, Singapore, Singapore

**Keywords:** COVID-19, immunology, pathology, diagnostics, specific T cells

## Abstract

In 2019, a novel coronavirus (SARS-CoV-2) was found to cause a highly contagious disease characterized by pneumonia. The disease (COVID-19) quickly spread around the globe, escalating to a global pandemic. In this review, we discuss the virological, immunological, and imaging approaches harnessed for COVID-19 diagnosis and research. COVID-19 shares many clinical characteristics with other respiratory illnesses.

Accurate and early detection of the infection is pivotal to controlling the outbreak, as this enables case identification, isolation, and contact tracing. We summarize the available literature on current laboratory and point-of-care diagnostics, highlight their strengths and limitations, and describe the emerging diagnostic approaches on the horizon.

We also discuss the various research techniques that are being used to evaluate host immunity in laboratory-confirmed patients. Additionally, pathological imaging of tissue samples from affected patients has a critical role in guiding investigations on this disease. Conventional techniques, such as immunohistochemistry and immunofluorescence, have been frequently used to characterize the immune microenvironment in COVID-19. We also outline the emerging imaging techniques, such as the RNAscope, which might also aid in our understanding of the significance of COVID-19-specific biomarkers, such as the angiotensin-converting enzyme 2 (ACE2) cellular receptor.

Overall, great progress has been made in COVID-19 research in a short period. Extensive, global collation of our current knowledge of SARS-CoV-2 will provide insights into novel treatment modalities, such as monoclonal antibodies, and support the development of a SARS-CoV-2 vaccine.

## Introduction

In December 2019, a novel respiratory disease named coronavirus disease 2019 (COVID-19) was detected by physicians in Wuhan, China. The disease was found to be caused by the severe acute respiratory syndrome (SARS)–CoV-2 RNA virus.^1,2^ Within a matter of weeks, COVID-19 had spread rapidly and escalated to a global pandemic. At the time of writing (June 2020), >10 million cases had been reported and >500,000 patients had succumbed to the disease worldwide.^3^ Indeed, patients with COVID-19 are at high risk of developing a severe and critical disease.^4^ Therefore, rapid and accurate diagnostic tests are urgently needed to effectively isolate, identify, and treat infected individuals and to contain the spread of the virus. Failure to do so will inevitably lead to spikes in cases and the resultant overcrowding and collapse of healthcare services.^5^ Moreover, research into this novel virus is also critical to understand its pathogenesis and its interaction with the human immune system. Insights from such research will guide the design of public health policies and protocols to identify susceptible individuals, and diagnostic, prognostic, and treatment approaches for patients.

Current diagnostic approaches predominantly involve established virological procedures, such as nucleic acid hybridization techniques (reverse-transcriptase PCR [RT-PCR]) and recombinase polymerase amplification (RPA), as well as immunologic approaches like antibody assays. Each approach boasts unique strengths and weaknesses. For instance, while RT-PCR demonstrates high sensitivity and specificity, its capabilities have been severely limited for practical reasons during this current pandemic due to global shortages of skilled personnel, reagents and equipment, and a processing time of up to 4 days. By contrast, immunologic tests, such as antibody assays, are rapid and require minimal equipment, but they have limited utility in the context of acute diagnosis of SARS-CoV-2 infections. This is because it can take several days to weeks following symptom onset for a patient to mount a detectable antibody response.^6^

Immunological tools in research include enzyme-linked immunosorbent assays (ELISAs), flow cytometry, and mass cytometry (CyTOF). Imaging techniques for pathological analyses include conventional approaches, such as hematoxylin–eosin (H&E) staining, immunohistochemical (IHC) staining, or transmission electron microscopy (TEM), and RNAscope. Each of these methods is used to examine the pathophysiology underlying COVID-19 from a different perspective, each with their own advantages and disadvantages. For example, it has been established that the entry of SARS-CoV-2 intro cells depends on the binding of viral proteins with the human receptor angiotensin-converting enzyme 2 (ACE2) receptors.^7^ Additionally, evidence shows that the type II transmembrane protease (TMPRSS2) is also essential for viral entry, by priming the viral spike protein for binding to ACE2.^8^ Therefore, considerable research efforts employing different techniques have been directed at mapping the distribution of ACE2 and TMPRSS2 in tissues and their relationship to the observed manifestations of disease. Together, the combination of these approaches has advanced our understanding of COVID-19.

In this review, we discuss the current approaches in COVID-19 diagnosis and research with a focus on findings from virological and pathological imaging methods. We also discuss immunological methods, which are increasingly recognized as an integral component of the disease process.

## Diagnostics

The most common symptoms of COVID-19 at initial presentation are nonspecific and include a high fever, a new and persistent cough, and fatigue.^9,10^ Due to similarities between the clinical characteristics of COVID-19 and many other respiratory illnesses, the accurate and early detection of infection is pivotal for outbreak control. Any delays in diagnosis are increasingly measured in lives lost.

According to the World Health Organization (WHO), the immediate goal for research into COVID-19 diagnostics is the development of RNA assays, antibody and antigen assays, and point-of-care detection.^11^ The intermediate-term priority would be their integration into multiplex diagnostic platforms, while the long-term goal would be the investigation of prognostic markers.

In this section, we summarize the current and emerging diagnostic tools for SARS-CoV-2 through the lens of immunology.

### Lab-Based Tests

#### RT-PCR Molecular Testing

The detection of viral nucleic acids by RT-PCR is the primary method used to confirm a suspected case of COVID-19. RT-PCR and other nucleic acid hybridization techniques are an integral part of virology and are applied in a broad range of settings, including screening, diagnosis, informing medical and therapeutic decisions, and assessing cure rates from therapy.^12^ Chinese officials released the genomic sequence of SARS-CoV-2 to public databases early in the course of the outbreak,^13^ and the WHO has since published seven protocols for RT-PCR-based diagnostics. Because of the high sensitivity and specificity of RT-PCR, it is regarded as the “gold standard” for virus detection.^14^ There are two essential steps in the process: (1) viral RNA extraction and (2) PCR amplification and probe-based detection. Multiple large-scale, high-throughput instruments are available for automating both steps, such as the Roche Cobas 6800 system, which has an advertised throughput of 1536 tests per 24 hours.^15^

However, RT-PCR-based testing is costly and time-consuming, requiring up to 4 days using centralized laboratory equipment and skilled personnel; furthermore, global supply chain challenges have led to significant shortages of essential reagents. Lastly, false-negative results due to low sample volumes, variable sampling techniques and sampling locations, sample degradation during transportation, and/or improper nucleic acid extraction are a concern.^16–18^ In addition, the differences in detectable viral material in different sampling locations (e.g., nasopharyngeal vs bronchoalveolar lavage fluid [BALF] vs rectal samples) might also explain the false-positive RT-PCR results on repeat testing in “recovered” COVID-19 patients. Indeed, one postmortem case study revealed residual virus in lung tissue despite consecutive negative results on PCR testing from nasopharyngeal swabs.^19^ Separately, Winichakoon et al. outlined a case of repeatedly negative nasopharyngeal and oropharyngeal swabs in a clinically deteriorating patient where only a BALF PCR test returned positive.^20^

Given the high expression of ACE2 on alveolar epithelial cells and negative expression on nasal, oral, and nasopharynx cells,^21^ it would be prudent to perform bronchoalveolar lavage on patients to rule out false-negative results from swabs of upper respiratory tract samples.^20^

#### Lab-Based Immunological Assays

In contrast to RT-PCR techniques that detect viral nucleic acids, serological and immunological assays aim to detect antibodies against SARS-CoV-2 or antigenic proteins in infected individuals. Neutralization assays are considered the gold standard for assessing neutralizing (protective) antibodies;^22^ however, these assays require specialized biosafety level 3 (BSL3) facilities and still take several days to complete. Another type of lab-based antibody assay is the traditional ELISA, which detects all binding antibodies. The four principal types of ELISA are direct, indirect, competitive, and sandwich ELISA; the indirect ELISA is the most common method for determining antibody concentrations. ELISAs have good concordance with neutralization assays for the detection of antibody responses in SARS-CoV-2.^23^ Unfortunately, both methods require skilled operators and are limited by low throughput due to the absence of fully automated systems.

Serological diagnostics offer several advantages. Re-quirements for specimen quality are comparatively less stringent than for nucleic acid tests as the antibodies are uniformly distributed in the serum.^24^ Consequently, sampling location concerns do not apply here. Furthermore, good correlation between IgG ELISAs performed on both conventional serum samples and plasma samples have been reported,^25^ of which the latter may be conveniently obtained from residual blood submitted for other routine laboratory tests.

One pitfall of antibody assays is their limited utility early in the course of any infection. Sparse data are available with regard to the antibody responses produced by patients with COVID-19. It seems that SARS-CoV-2 IgM is detectable at a median of 5 days after symptom onset, while IgG is detectable after 14 days,^26^ with the seroconversion rate approaching 100% by day 39. An Italian research group noted that the performance of a commercial VivaDiag COVID-19 IgM/IgG test was very poor, with a sensitivity of only 18.4% and a negative predictive value of 26.2% in a cohort of suspected COVID-19 patients in the emergency room setting.^27^ As such, we believe that for now RT-PCR testing is likely more appropriate for diagnosing acute COVID-19.

Notably, a longitudinal study examining the IgG/IgM profiles of 63 patients found that seroconversion for IgG and IgM occurred in no specific chronological order, with a median of 13 days after symptom onset;^28^ all patients achieved seroconversion by day 20. Consequently, the detection of both IgG and IgM simultaneously rather than one antibody alone would be ideal.

Another concern surrounding serologic diagnostics is the production of false-positive results from cross-reactivity, due to the high prevalence of the four endemic human coronaviruses in the human population. In SARS-CoV-2, the spike (S) protein (which includes two regions, S1 and S2) and the nucleocapsid (N) protein (NP) are the major immunogens,^29^ and therefore most diagnostics rely on the detection of antibodies specific for these antigens. One work suggests that of the possible targets, the S1 subunit antigen is more specific than either the whole S antigen or the N antigen for detecting SARS-CoV-2 antibodies, with no cross-reactivity to other coronaviruses except for SARS-CoV.^23^ Given that only 8096 SARS-CoV infections were recorded worldwide,^30^ the risk of false positives from this cross-reactivity is miniscule. However, NP ELISAs are more sensitive than S1 in detecting antibodies in those with a mild infection.^23^ Importantly, as in SARS-CoV, most of the neutralizing antibodies are directed against the S protein,^31^ of which S1 contains a receptor-binding domain (RBD) responsible for making contact with ACE2 to facilitate viral entry.^7^ Thus, theoretically only diagnostics that detect S1-specific antibodies are suitable to infer immunity to COVID-19; this fact is corroborated by evidence that anti-S RBD, but not anti-NP IgG levels correlated with neutralizing antibody titers in sera from a cohort of 14 recovered patients.^32^ The number of commercial antibody assays is growing, detecting either anti-NP antibodies, anti-S1/S antibodies, or both; there is also large variation in their claimed sensitivities and specificities.^33^ Based on the available evidence, an ideal serological assay would be a combined test that simultaneously detects both antibodies to NP and S1 antigens; assessment of anti-NP antibodies has good sensitivity and would be best suited for supporting the diagnosis of infection, while the additional anti-S1 antibody assay would allow for the determination of immunity.

### Rapid Tests

#### Point-of-Care RT-PCR Tests

A small number of commercial point-of-care tests utilizing RT-PCR have been developed. These typically involve the same methodology as conventional RT-PCR, but implemented with automated and portable benchtop-sized instruments that can be operated closer to patient care settings than a centralized laboratory. A prominent example is Cepheid’s Xpert Xpress SARS-CoV-2, run on the Gene Xpert platform. This apparatus can provide a result within 45 min. Others include the MesaBioTech Accula Test and MicrosensDx RapiPrep COVID-19. Despite displaying good sensitivity and specificity, these instruments are generally limited by a very low throughput of only one to four tests per run per machine^34^ and, as such, are only suited to small laboratories or clinics.

#### Immunological Assays

##### Rapid Antibody Assays

**Figure 1.**
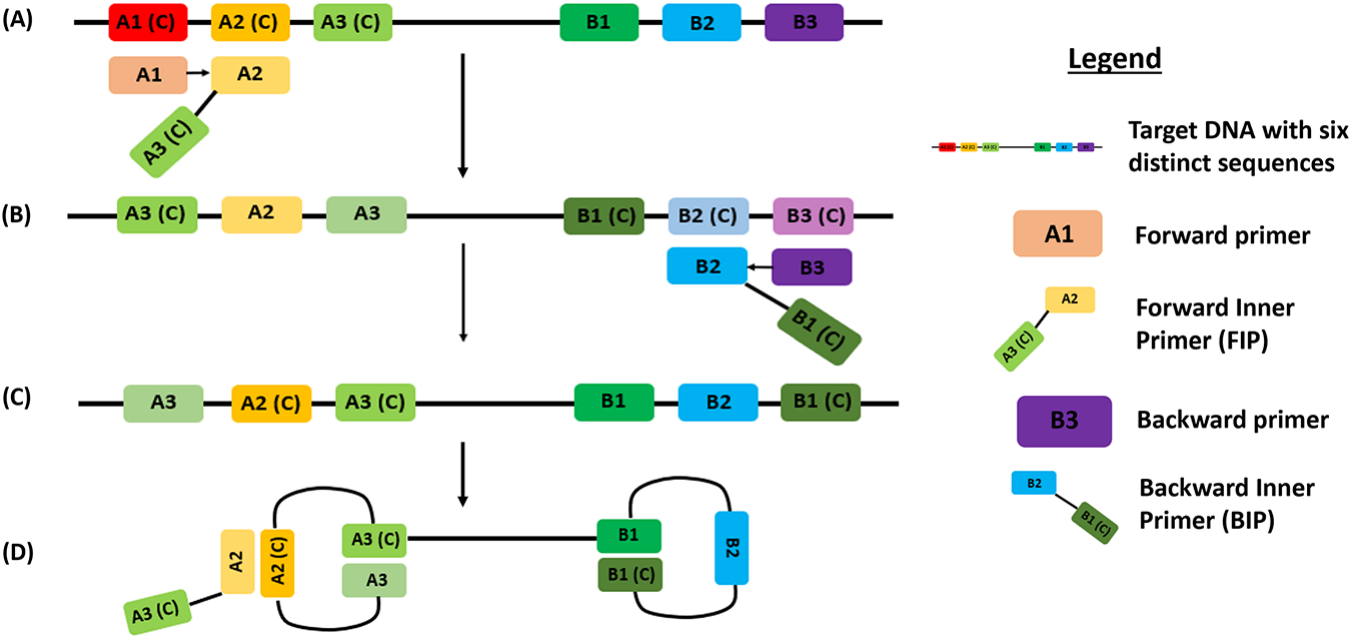
Loop-mediated isothermal amplification (LAMP). (**A**) LAMP begins when the forward inner primer (FIP) binds to the A2(C) region while the forward primer (A1) binds to A1(C), which displaces the FIP complementary strand. (**B**) The backward inner primer (BIP) binds B2(C) while the backward primer (B3) binds B3(C) and displaces the BIP complementary strand. (**C**) A complementary sequence that initiates loop formation is produced. (**D**) Loop structures are formed that allow for LAMP with the use of loop primers.

**Figure 2.**
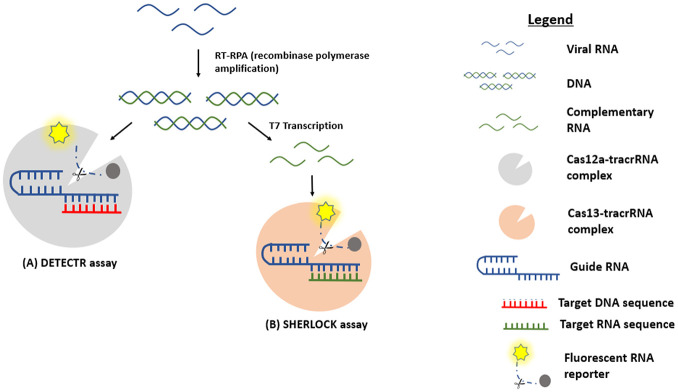
CRISPR technique. Viral RNA is converted to dsDNA using RT-RPA (recombinase polymerase amplification). (**A**) The CAS12a nuclease enzyme is activated upon complex binding to the target sequence, resulting in cleavage of the target sequence and the fluorescent RNA reporter. (**B**) T7 transcription converts DNA to complementary RNA. Cas13 nuclease enzyme activity is activated upon complex binding to the target sequence, resulting in a similar cleavage of the target sequence and the fluorescent RNA reporter.

Compared with lab-based antibody assays, rapid assays such as lateral flow immunoassays (LFIAs) (**Fig. 1**) and chemiluminescent immunoassays (CLIAs) (**Fig. 2**) offer the benefits of rapid diagnostic testing at a low cost. These assays do not require specialized equipment or expertise^35^ and are thus excellent candidates for point-of-care testing or deployment on a large scale. This an area of intense interest, with governments worldwide aiming to order millions of tests to inform policy makers about attack rates in their populations.^36^ LFIAs are predominantly single-use kits designed for point-of-care use, while CLIAs are fully automated analyzers that permit very high testing throughput.

Unfortunately, these tests do not quantify the antibody titers, and the performance of LFIAs has been called into question; one evaluation of nine commercial LFIAs reported a sensitivity ranging from only 55% to 70% versus RT-PCR and 65% to 85% versus ELISA.^37^ Meanwhile, the performance of CLIAs is superior, with good sensitivity and specificity levels similar to those of ELISA.^38^ Otherwise, these tests share the same advantages and drawbacks as the lab-based antibody assays discussed above. The characteristics and unique advantages and disadvantages of these different methodologies are outlined in **Table 1**.

**Table 1.** Summary of Diagnostic Approaches for COVID-19.

Category	Type of Test	Typical Test Result Time	Characteristics	Examples
Virologic/molecular tests	RT-PCR	Days	Gold standard, high sensitivity and specificity, high throughput but lab based	WHO RT-PCR protocols
	Point-of-care RT-PCR	30–45 min	Rapid, good sensitivity and specificity, point-of-care testing but low throughput	Cepheid Xpert Xpress SARS-CoV-2
	LAMP, CRISPR	<1 h	Rapid, good sensitivity and specificity, point-of-care testing but low throughput	Sherlock Biosciences SHERLOCK
Immunologic tests	LFIA (for antibodies/antigens)	15–20 min	Rapid, point-of-care testing but not quantitative, poor sensitivity	VivaDiag COVID-19 IgM/IgG rapid test Corisbio COVID-19 Ag Respi-Strip
	Traditional ELISA	2–5 h	Good sensitivity and specificity but lab based, not automated	Epitope Diagnostics KT-1033 EDI Novel Coronavirus COVID-19 ELISA kit
	CLIA	30 min	Rapid, good sensitivity and specificity, high throughput but lab based	Roche Elecsys Anti-SARS-CoV-2
	Neutralization assay	Days	Gold standard, high sensitivity and specificity, able to quantify neutralizing antibodies but requires BSL-3 lab facility	Not commercially available

##### Antigen Assays

An alternative approach to immunological assays is to directly detect SARS-CoV-2 viral antigens. Several commercial point-of-care antigen tests are available, but their performance remains to be evaluated. These tests may be suitable for making an early diagnosis and are deployable as point-of-care assays. However, they face the same sampling limitations as RT-PCR and are hypothetically hampered by limited sensitivity due to the omission of an amplification process, unlike nucleic acid testing. For example, one multicenter study evaluating the Corisbio COVID-19 Ag Respi-Strip, a lateral flow assay for the SARS-CoV-2 NP, reported a test sensitivity of only 57.6%.^39^

#### Rapid Non-PCR Molecular Testing

Nucleic acid testing using non-PCR methods is an emerging approach for rapid diagnostics, and several assays have received Food and Drug Administration (FDA) emergency use authorization, which facilitates the distribution of unapproved medical products, or the off-label use of approved medical products when certain criteria are met. These methods share high sensitivity and specificity on par with RT-PCR, but with the principal advantages of more rapid testing at a lower cost.^40–42^

**Figure 3.**
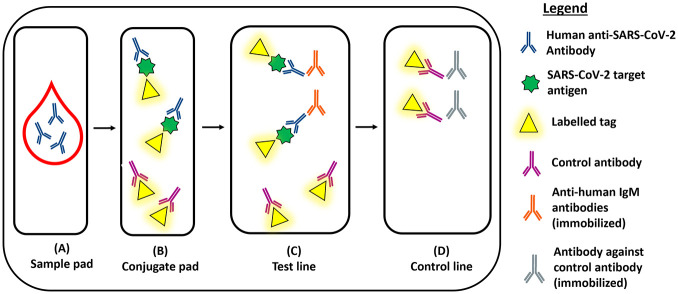
Lateral flow immunoassay (LFIA). (**A**) Serum sample deposited on the sample pad. (**B**) Anti-SARS-CoV-2 antibodies in the sample will bind to the target antigen with a labeled tag. (**C**) Immobilized anti-human IgM antibodies will capture the SARS-CoV-2 antibody–antigen complex. (**D**) Control antibodies are captured by immobilized antibodies in the control line.

LAMP (**Fig. 3**) is one such novel isothermal nucleic acid amplification method that does not require a thermal cycler. One example is the ID NOW COVID-19 test from Abbott Diagnostics, which can deliver results in just 5 min^43^ and uses a lightweight portable instrument, allowing on-site testing of swab samples. However, it has a limited throughput of only one sample per run.

**Figure 4.**
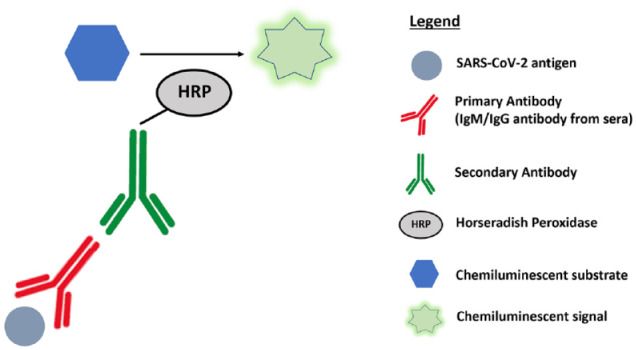
Chemiluminescence enzyme immunoassay (CLIA). SARS-CoV-2 antigens will capture IgM and IgG antibodies from the sample serum. Secondary antibodies that are conjugated with horseradish peroxidase (HRP) bind to the captured primary IgM and IgG antibodies and react with a chemiluminescent substrate to generate a strong chemiluminescent signal that is measured in terms of relative light units (RLU).

The CRISPR enzymes Cas12 and Cas13 have also been adapted for rapid nucleic acid sensing ^44^ (**Fig. 4**). The DETECTR assay by Mammoth Biosciences,^45^ as well as the SHERLOCK assay by Sherlock Biosciences,^46^ potentially offers sensitivity and specificity comparable to those of RT-PCR, but can be completed in ~1 h. However, these approaches are still in the early stages of commercialization and current applications are available only as test kits to be run in labs, while point-of-care versions exist as proof-of-concept demonstrations.^47^ Nonetheless, their inherent characteristics hold great potential for diagnosis in the future.

### Prognostication of Disease

#### Profiling of Genetic Susceptibility

Work is in progress to ascertain the possible genetic basis for the apparent variations in COVID-19 susceptibility and disease severity. Cao et al. compared expression quantitative trait loci (eQTL) for ACE2 in different populations, finding significantly greater eQTL variants associated with higher ACE2 expression in East Asian populations, but reported no direct evidence supporting the existence of S protein-binding-resistant ACE2 mutants^48^ out of 32 identified protein altering variants. Separately, Stawiski et al. analyzed ACE2 polymorphisms within a much larger population dataset spanning more than 400 population groups across the world and performed structural predictions to identify variants that might confer protection or rather increase susceptibility to SARS-CoV-2 S protein binding.^49^ Out of a total of 298 identified protein-altering ACE2 variants, 14 variants were predicted to increase susceptibility while 26 variants were speculated to confer protection; however, the degree of changes in receptor–virus binding interactions for each structural variant was not quantified. These findings represent significant developments in our understanding of population risk profiles for COVID-19 and future coronavirus infections.

#### Serum Prognostic Markers

Another application of immunological methods would be to measure markers that enable prognostication in COVID-19. Higher titers of antibodies against SARS-CoV-2 have been associated with more severe disease,^23,50^ similar to previous studies in Middle East respiratory syndrome (MERS)–CoV.^51^ ELISA has been used to provide a quantitative measurement of serum and plasma IgM and IgG antibodies. By monitoring the kinetics of IgM and IgG antibodies specific to the N and S proteins on SARS-CoV-2, it was found that intensive care unit (ICU) patients had a significantly lower level of S-IgG within 2 weeks of symptom onset but a higher level of N-IgG antibodies compared with non-ICU patients.^52^ This finding highlights the possible utility of S-IgG and N-IgG as a prognostic tool for COVID-19 patients.

The D-dimer level, which consists of cross-linked fibrin degradation products that reflect ongoing blood clot formation and breakdown activity in the body, is another proposed prognostic marker. Modern commercial assays for D-dimers are based on monoclonal antibodies, employing either ELISA or microlatex agglutination assays.^53^ Reports have emerged that elevated D-dimer levels, suggestive of a hypercoagulable state, are associated with drastically worse outcomes. A Chinese group reported that D-dimer levels of ≥2.0 µg/mL on admission were associated with a 51.5 times increased mortality relative to D-dimer levels of <2.0 µg/mL in a cohort of 343 COVID-19 patients.^54^ This finding of D-dimer levels as a negative prognostic marker was also noted in other studies conducted in China^4,55^ and the Netherlands.^56^

Similarly, interleukin (IL)-6, a key component of the cytokine release syndrome, is another marker measured by ELISA and has been described to independently predict adverse outcomes in COVID-19.^57,58^ Tumor necrosis factor alpha (TNFα), another important pro-inflammatory cytokine, has also been found to be strongly correlated with end-organ damage and mortality even after adjusting for disease severity scores.^59^ Gao et al. examined both IL-6 and D-dimer levels; they proposed a panel comprising tandem testing of these two markers, which produced a sensitivity of 96.4% and specificity of 93.3% in early prediction of severe COVID-19.^58^ Elevated troponin levels, a marker of myocardial injury measured with ELISA immunoassays, also strongly predict progression to death in patients with severe illness.^60^ These results suggest that multiplex cytokine and serum marker profiling will be a powerful tool in stratifying patients that may guide clinical decisions and resource allocation.

##### Summary

In sum, rapid progress has been made in diagnostics for COVID-19. Yet the race against time continues for researchers and biotechnology firms to develop rapid, cost-effective, and reliable test kits that can be deployed on a large scale. At the time of writing, lab-based RT-PCR testing has been the dominant diagnostic approach, but alternative molecular approaches like isothermal amplification and CRISPR, which have clear advantages, are on the horizon. Immunological tests such as CLIA and LFIA will become increasingly important because of the urgent need for point-of-care diagnostics for mass testing of infected asymptomatic individuals and their close contacts, and will be valuable in complementing molecular approaches for confirming infection. Furthermore, immunological assays will be in great demand by policy makers worldwide for the assessment of immunity to COVID-19. However, the performance of these serological tests varies significantly, particularly their degree of sensitivity and specificity; we believe that caution must be taken in the interpretation of these tests. Detailed evaluation of the reliability of serological tests will be a key area for future research. Lastly, given the importance of techniques like ELISA in prognosticating COVID-19, immunological methods will undoubtedly occupy a crucial role in achieving all levels of the WHO’s short-, medium-, and long-term diagnostic goals.

## COVID-19 Research Tools

### Immunological Approaches

COVID-19 infection has a poor prognosis in individuals with comorbidities and abnormal immune functions. Although research surrounding COVID-19 is still in its infancy, several studies have revealed lymphopenia and the cytokine storm as underlying mechanisms correlating to disease progression. Here, we discuss the various immunological techniques involved in assessing host immunity in COVID-19 patients.

#### ELISA

As discussed, ELISA has also been used to detect the inflammatory cytokines implicated in the cytokine storm seen in patients with severe respiratory failure due to COVID-19. One study found that the immune dysregulation in patients with severe respiratory failure was due to a significantly increased production of IL-6 and defective lymphoid function because of an IL-6-mediated decrease in HLA-DR expression on CD14 monocytes. Interestingly, interferon-gamma (IFNγ) levels were below the detection level in these patients, suggesting that T helper (T_h_) 1 cells are unlikely to be major players in the overinflammatory response of severe patients.^61^ A similar observation was made in a separate study whereby inflammatory cytokines that mediate major immune responses, such as TNFα and IL-1β, were not significantly elevated in ICU patients.^62^ These findings demonstrate that the immunophenotype of patients with COVID-19 can vary depending on presently unclear host immune factors and the severity of their condition. This relationship between disease severity and cytokine storm has also been highlighted in other studies that found a significantly elevated plasma concentration of granulocyte colony-stimulating factor (G-CSF), IP10, CCL2, and CCL3 in ICU patients compared with non-ICU patients.^63^

ELISA is also being used as a companion diagnostic tool for therapeutic purposes. In a study that explored the use of convalescent plasma therapy from donors as a form of treatment in severe COVID-19, ELISA was used to assess the neutralizing activity of the RBD-specific IgM and IgG antibodies found in the donor convalescent plasma.^64^ After the transfusion was complete, ELISA was also used to detect IgG, IgM, and neutralizing antibody titers in the sera of patients to assess the response to treatment.^65^

#### Enzyme-Linked Immunosorbent Spot

Enzyme-linked immunosorbent spot (ELISPOT) is a sensitive immunoassay that quantitatively measures cytokine-secreting cells at the single-cell level, providing insight into immune-related cellular activities.^66^ Hence, it is a promising tool for characterizing specific T-cell immunity in COVID-19 patients. By IFNγ ELISPOT analysis, it was revealed that convalescent COVID-19 patients had significantly increased levels of IFNγ-secreting T cells when compared with healthy donors. A significant correlation between neutralizing antibody titers and NP-specific T cells was identified in these patients, suggesting that a combination of humoral and cellular immunity is integral to clearing SARS-CoV-2. Interestingly, it was noted that in convalescent patients 2 weeks postdischarge, IFNγ-secreting T-cell numbers had decreased, suggesting that they may not be maintained for a prolonged period of time even in recovered patients.^67^

ELISPOT is also serving a vital role in vaccine development through the detection of potential T-cell epitopes in the S protein RBD of SARS-CoV-2.^68^ One study was able to harness ELISPOT assays to identify three T-cell epitopes that induced a strong adaptive immune response postimmunization, demonstrating the promise of ELISPOT assays in the area of vaccine development.^32^ Recently, ELISPOT has also been applied to assess the immunogenicity of newly developed vaccines. One such study successfully utilized an IFNγ ELISPOT assay to evaluate T-cell responses to a new SARS-CoV-2 vaccine in murine splenocytes and rhesus macaque peripheral blood mononuclear cells (PBMCs). The promising findings from this animal study informed the start of a phase I clinical trial with the same vaccine, highlighting the usefulness of ELISPOT in assessing immune responses to new vaccines and promoting vaccine development.^69^

#### Flow Cytometry

Unlike ELISA and ELISPOT, flow cytometry determines the number of cytokine-secreting cells and has the capacity to immunophenotype based on surface and intracellular markers.^70^ In relation to the current pandemic, this technique enables the detection, sorting, and analysis of multiple subpopulations of immune cells specific to COVID-19. Using flow cytometry, researchers detected a cytotoxic immune environment in patient blood samples, despite a reduction in the overall lymphocyte population.^71–73^ As part of the SARS-CoV-2 antiviral response, peripheral lymphocytes retain the capacity to activate and differentiate into subpopulations, such as antibody-secreting cells (CD3^–^CD19^+^CD27^hi^CD38^hi^), follicular T cells (CD4^+^CXCR5^+^ICOS^+^PD1^+^), CD4 T_h_ cells (CD38^+^HLA-DR^+^CD4^+^), cytotoxic T (T_c_) cells (CD38^+^HLA-DR^+^CD8^+^), and regulatory T (T_reg_) cells (CD3+CD4+CD25+CD127–).^71,72,74^ These T_c_ cells harbor large amounts of cytotoxic granules, while CD4 T_h_ cells skewed toward a pro-inflammatory T_h_1 and T_h_17 phenotype.^72,73,75^ The overall hyperinflammation and cytotoxic environment supports the notion that a cytokine storm could be liable for the multisystemic insults in patients with severe COVID-19.

Elicitation of antiviral T-cell responses specific to SARS-CoV-2 is of utmost importance to establishing viral control. Many studies have demonstrated robust antiviral responses; however, there is no known set of markers reported to identify SARS-CoV-2-specific T cells. Collectively, most groups have characterized SARS-CoV-2-specific T cells based on HLA-DR, CD38, CD69, CD25, CD44, and Ki-67 expression. These activated T cells are then responsible for the excessive secretion of inflammatory cytokines IL-2, IL-6, IL-8, TNFα, IFNγ, granulocyte-macrophage colony-stimulating factor (GM-CSF), granzyme B, and perforin.^68,72–78^ Upon SARS-CoV-2-peptide stimulation, peptide-specific T cells were identified based on the upregulation of HLA-DR, CD38, OX40, CD69, and CD137.^75–77^ Subpopulations were then identified based on CD45RA and CCR7 expression.^75^

It is also plausible that prolonged infection could induce lymphocyte reduction and functional exhaustion, which therefore mediates disease progression. T cells from COVID-19 patients have markedly higher expression of PD-1 and Tim-3 and produce significantly higher levels of TNFα, IL-6, and IL-10; such upregulation of PD-1 and Tim-3 positively correlates with disease severity in patients, while elevated levels of cytokines negatively correlate with T-cell counts.^78,79^ Additionally, high NKG2A expression was also identified on natural killer (NK) cells and T_c_ cells of COVID-19 patients. These cells also displayed reduced CD107a, IFNγ, IL-2, granzyme B, and TNFα secretion. Relative to convalescent patients, the frequency of NKG2A-expressing cells is higher in patients, which further supports the positive correlation between immune cell exhaustion and disease progression.^80^ When comparing mild and severe cases of infection, the latter seem to display more significant dysregulation of host immunity.^71^ Hence, it is evident that the effects of COVID-19 on lymphocytes, particularly T cells, ultimately disrupt host antiviral immunity (**Table 2**).

**Table 2.** Comparison of T-Lymphocyte Subsets between Mild and Severe Cases of COVID-19 Infection.^a^

Subset	Mild Cases	Severe Cases
CD4^+^ **T cells** **(CD3**^+^**, CD4**^+^**)**	↓	↓↓
CD8^+^ **T cells** **(CD3**^+^**, CD8**^+^**)**	↓	↓
Naïve CD4^+^ **T cells** **(CD3**^+^**, CD4**^+^**, CD45RA**^+^**)**	Normal	Normal; higher than mild cases
Memory CD4^+^ **T cells** **(CD3**^+^**, CD4**^+^**, CD45RO**^+^**)**	Normal	Normal; lower than mild cases
Suppressor CD8^+^ **T cells** **(CD3**^+^**, CD8**^+^**, CD28**^+^**)**	Normal	Normal; lower than mild cases
Activated T cells **(CD3**^+^**, HLA-DR**^+^**)**	Normal	Normal
Activated CD8^+^ **T cells** **(CD3**^+^**, HLA-DR**^+^**, CD8**^+^**)**	Normal	Normal
T**_reg_ cells** **(CD3**^+^**, CD4**^+^**, CD25**^+^**, CD127**^lo^**)**	↓	↓↓
**Naïv**e T**_reg_ cells** **(CD45RA**^+^**, CD3**^+^**, CD4**^+^**, CD25**^+^**, CD127**^lo^**)**	↓	↓↓
Induced T**_reg_ cells** **(CD45RO**^+^**, CD3**^+^**, CD4**^+^**, CD25**^+^**, CD127**^lo^**)**	↓	↓↓

a Interpreted from lymphocyte subset analysis of Qin et al.^71^ The down arrow (↓) indicates below normal range; the number of arrows reflects degree of reduction.

#### Mass Cytometry

CyTOF is another research tool that is being explored in the fight against COVID-19. This technique is a variation of flow cytometry that uses metal isotopes conjugated to antibodies to analyze specific cellular antigens. One CyTOF-based analysis of peripheral blood samples from COVID-19 patients found that those with severe disease had a significantly decreased number of CD4^+^ and CD8^+^ T cells compared with normal controls.^81^ Another CyTOF-based study demonstrated that COVID-19 patients with mild disease severity had increased proportions of dendritic cells, macrophages, CD4^+^ T cells, and TGFβ^+^CD28^–^ naïve CD8^+^ T cells when compared with those with severe disease.^82^ Hence, by profiling multiple immune cellular components, CyTOF can elucidate the immune response and disease progression in COVID-19 patients. CyTOF has also been used to assess the efficacy of emerging forms of treatment for COVID-19, including mesenchymal stem cell transplantation, by precisely characterizing the different subsets of immune cells such as CXCR3^+^CD4^+^ T cells, CD8^+^ T cells, and NK cells.^83^ Another CyTOF study identified that CD11c myeloid dendritic cells and CCR6^+^ T cell subtypes were the most vulnerable to IL-1β-induced inflammatory signaling. These data might permit the development of therapeutics targeting the IL-1R, which has been implicated in the cytokine storm often observed in severe COVID-19 patients.^84^

#### Single-Cell RNA Sequencing

Single-cell RNA sequencing (scRNAseq) has been widely used to explore gene expression profiles at the cellular level. For example, BALF has been analyzed by scRNAseq to characterize the landscape of the lung immune microenvironment in both mild and severe cases.^85^ Patients of age and/or with comorbidities are more susceptible to poor prognosis. One possible contributory factor, as has been observed in SARS-CoV, is age-related dendritic cell function in the lung immune microenvironment, which resulted in impaired migration to draining lymph nodes and hence aberrant immune responses toward the disease.^86^ Similarly, sequencing analyses of BALF from COVID19 patients revealed that mild cases have a higher proportion of NK cells and CD8 T cells but lower levels of inflammatory macrophage recruitment compared with severe cases. Infiltration of highly inflammatory monocyte-derived FCN1^+^ macrophages was observed in severe cases.^85^ Perhaps dysregulation of macrophage and lymphocyte populations in the lungs is a contributing factor to the observed lung function failure.

The landscape of the blood immune microenvironment during recovery stages has also been determined. Discharged patients in their early recovery stages seem to exhibit a hyperinflammatory immune signature and a significantly higher population of CD14^+^IL-1β^+^ monocytes. The scRNAseq data suggested that monocyte activation and proliferation was mediated by B-cell-derived IL-6, T cell-derived macrophage colony-stimulating factor (M-CSF), and GM-CSF. As host immunity lingers in a hyperinflammatory state, it accounts for patients who have been discharged returning to the hospital feeling unwell.^87^ Apart from consistent reports of lymphopenia, the use of scRNAseq has thus provided novel insights into the role of monocytes in COVID-19 progression.

One complication of COVID-19 is acute kidney injury.^88^ Utilizing scRNAseq, ACE2 receptors and TMPRSS proteases were found to be coexpressed in podocytes and proximal straight tubule cells.^89,90^ Successful identification of candidate host cells in the kidney, along with proteinuria reported in COVID-19 patients, highlights the importance of monitoring patient renal function.^88,89^ In another instance, coexpression of ACE2 and TMPRSS was found in gastrointestinal tissue, including in the upper epithelial and gland cells of the esophagus, and absorptive enterocytes from the ileum and colon.^91^ This finding, together with one report of detectable live virus in fecal specimens from Wang et al., suggests fecal–oral transmission should be considered a possible route of transmission.^92^ Because the target cell type of SARS-CoV-2 remains unclear, it is crucial to identify ACE2 and TMPRSS coexpressing cells to improve disease management strategies.

Thus far, research efforts have largely focused exclusively on the characterization of COVID-19 in adult patients, and sparse data exist regarding its implications in developing embryos. Notably, scRNAseq analysis has suggested that human embryos are also susceptible to the virus.^93,94^ The possibility of vertical transmission is relatively high since *ACE2* and *TMPRSS2* are both expressed within cells of the cytotrophoblast and syncytiotrophoblast in the placenta, as well as the epiblast cells of human embryos.^93^ Interestingly, genes involved in the novel ACE2-independent route of entry, which utilizes the basigin (BSG) receptor, also known as *CD147*, as well as an endosomal protease involved in processing of the SARS-CoV-2 S protein for viral entry, cathepsin L (*CTSL*), were also detected throughout all stages. Furthermore, cells in the inner cell mass, epiblast, and primitive endoderm were also found to express the majority of the genes required for viral endocytosis and replication.^94^ As the pregnancy progresses, specific cell types of the fetal heart, liver, and lung begin to coexpress *ACE2* and *TMPRSS2* as well.^93^ Although further studies are warranted, the fact that (1) ACE2 and TMPRSS2 are coexpressed on cells in the maternal–fetal interface and the epiblast and that (2) CD147 and CTSL are coexpressed in the majority of embryonic cells suggests that it is advisable to avoid pregnancy during this pandemic due to the potential for maternal–fetal transmission of COVID-19. As cells of the epiblast undergo organogenesis, it is difficult to exclude the possibility that SARS-CoV-2 infection in early gestation may result in organ malformation or even fetal mortality.

##### Single-Cell TCR Sequencing and Single-Cell BCR Sequencing

Genes encoding the T-cell receptor (TCR) and B-cell receptor (BCR) are composed of variable (V), diversity (D), and joining (J) segments. With somatic recombination occurring during T-cell development, it gives rise to an extensive number of T-cell repertoires with different antigen-binding abilities.^95^ Thus, another method to evaluate the T-cell response is through its clonal expansion, using single-cell TCR sequencing (scTCRseq). Sequencing analyses of T cells isolated from the BALF of COVID-19 patients have shown that ZNF683^+^CD8^+^ T cells have the highest clonal expansion level and CCR7^+^ central memory T cells have the lowest.^85,87^ In mild cases of COVID-19, researchers observed significantly higher expansion levels of total T cells and ZNF683^+^CD8^+^ T cells, implying potential specificity to SARS-CoV-2.^85^ Patients in the early recovery stages have significantly reduced T-cell expansion levels, with the expanded CD8^+^ T-cell clones exhibiting excessive inflammation and antiviral activity.^87^ Overall, these findings support the involvement of CD8^+^ T cells in resolving SARS-CoV-2 infection.

With the intense emphasis on T-cell responses, B-cell responses have been relatively overlooked. Yet in response to SARS-CoV-2 infection, antibody-secreting cells are activated and serum immunoglobulins levels are elevated.^72,87^ During the process of B-cell development to plasma cells, somatic hypermutation occurs to generate high-affinity antibodies. In COVID-19 patients, significant increases in plasma cell counts and a notable bias in genes that underwent unique VDJ rearrangements have been reported.^87^ Further single-cell BCR sequencing (scBCRseq) analysis of B cells from these COVID-19 patients in early recovery stages revealed that CD27^+^CD38^+^ memory B cells have the highest clonal expansion levels, while IL-4R^+^ naïve B cells have the lowest levels. The expanded B-cell clones are predominantly IgA and IgM isotypes. Despite this novel identification of BCR signaling, further studies are needed to assess the precise role of humoral immunity in COVID-19 pathogenesis.

#### Key Areas for Further Research

While the cellular entry of SARS-CoV-2 has consistently been reported to be mediated by ACE2,^7^ specific immune cell targets remain unclear. One pseudovirus infection study on T-lymphocyte cell lines demonstrated the ability of SARS-CoV-2 to infect T cells through receptor-dependent, S protein-mediated membrane fusion.^96^ This finding is surprising, as these cell lines have low ACE2 expression, and flow cytometry analysis suggests an increase in susceptibility to SARS-CoV-2 over time. It was subsequently discovered that SARS-CoV-2 failed to replicate in these infected T cells and was subject to viral RNA degradation.^96^ However, another report observed an absence of detectable viral NP antigen in CD33^+^ T cells and B220^+^ B cells.^97^ Considering the conflicting evidence, further work is needed that ideally uses patient samples and viral cultures. Separately, other researchers have proposed novel receptor routes (e.g., via CD147) for viral entry and infection.^98^

As COVID-19 remains an ongoing pandemic, little is known about the extent of host memory immune responses. Notably, in a 6-year follow-up study on SARS patients, the maintenance of SARS-specific anamnestic memory T-cell responses was identified despite undetectable levels of memory B cells and SARS-specific IgG.^99^ These findings are instructive for preparations against disease reemergence. Moving forward, we urge researchers to conduct similar follow-up studies on COVID-19 patients as well, with the aim of identifying novel treatment targets and assessing the maintenance of protective immunity in recovered patients.

#### Summary

Intense research involving immunological approaches (including ELISA, CyTOF, and scRNAseq) has resulted in a preliminary understanding of the inflammatory immunopathogenesis of COVID-19 and uncovered even more potential areas for investigation. However, the full picture of the pathophysiology of this disease is still poorly defined. An overview of identified alterations in immune cell subsets, cytokines, and gene expression upregulation in COVID-19 is provided in **Table 3**. The aforementioned techniques are tremendously valuable in disease management and surveillance due to their capacity to analyze immune cells and cytokines in a microenvironment. Of those discussed, ELISA holds great promise in the field of diagnostics due to its ability to detect IgM and IgG antibodies targeted at the S and N proteins of SARS-CoV-2. While rapid IgM and IgG diagnostic tests fare poorly in the early phase of disease,^100,101^ they are indispensable tools in detecting asymptomatic and recovered individuals. Serological testing using ELISA has been used in epidemiological investigations to successfully identify asymptomatic carriers who might serve as nodes of transmission, allowing for improved contact tracing and containment efforts.^102^ As countries begin to lift lockdown measures, ELISA may also potentially be used to track seroprevalence and herd immunity within a region of interest to guide reopening measures.^103,104^

**Table 3.** Summary of Identified Immune Cell Subsets, Cytokines, and Gene Expression Upregulation in Comparison with Healthy Controls.^a^

**Authors**	Phase of Infection	Sample Type Technique	Immune Cell Subset, Cytokines, **and Gene Expression**
Giamarello**s-Bourboulis** et al.^61^	Acute	Blood ELISA	CD4^+^ T cells (IL-6 and IFNγ^) CD14 monocytes (IL-6)
Wei et al.^62^	Acute	Blood ELISA	Monocytes (TNF and IL-1β)^
Huang et al.^63^	Acute	Blood ELISA	Laboratory findings on cytokine levels: IL-1B, IL-1RA, IL-7, IL-8, IL-9, IL-10, basic FGF, G-CSF, GM-CSF, IFNγ, IP10, CCL2, CCL3, CCL4, PDGF, TNFα, and VEGF Laboratory findings on cytokine levels (in ICU patients compared with non-ICU patients): IL-2, IL-7, IL-10, G-CSF, IP10, CCL2, CCL3, and TNFα
Ni et al.^67^	Convalescent	Blood ELISPOT	NP-specific T cells RBD of S protein-specific T cells (IFNγ secretion increased in convalescent patients)
Shi et al.^74^	Acute	Blood flow cytometry	CD3^+^CD8^+^ T cells* CD3^+^CD4^+^ T cells* (IL-2, IL-4, IL-6, and TNFα) CD3^+^CD4^+^CD25^+^CD127– Treg cells (IL-10) CD3–CD19^+^ B cells* CD3–CD16^+^CD56^+^ NK cells*
Qin et al.^71^	Acute	Blood flow cytometry	CD3^+^CD4^+^ T cells* CD3^+^CD8^+^ T cells* CD45RA^+^CD3^+^CD4^+^CD25^+^CD127^lo^ naïve Treg cells* CD3^+^CD4^+^CD25^+^CD127^lo^ Treg cells* CD45RO^+^CD3^+^CD4^+^CD25^+^CD127^lo^ induced Treg cells* CD3^+^CD19^–^ B cells* Laboratory findings on cytokine levels: IL-1β, IL-2, IL-6, IL-8, IL-10, TNFα, IFNγ
Thevarajan et al.^72^	Acute	Blood flow cytometry	CD38^+^HLA-DR^+^CD8^+^ T cells (granzyme A, granzyme B, and perforin) CD38^+^HLA-DR^+^CD4^+^ T cells CD4^+^CXCR5^+^ICOS^+^PD1^+^ follicular T cells CD3^–^CD19^+^CD27^hi^CD38^hi^ antibody-secreting cells CD16^+^CD14^+^ monocytes*
Xu et al.^106^	Patient deceased	Blood flow cytometry	CD38^+^HLA-DR^+^CD4^+^ T cells CCR6^+^CD4^+^ T_h_17 cells CD38^+^HLA-DR^+^CD8^+^ T cells (perforin and granulysin)
Braun et al.^76^	Acute	Blood flow cytometry	CD38^+^HLA-DR^+^CD8^+^ T cells CD4^+^CD40L^+^CD137^+^ T cells CD38^+^HLA-DR^+^CD4^+^Ki-67^+^ T cells
Weiskopf et al.^75^	Acute	Blood flow cytometry	CD45RA^+^CCR7^+^ naïve T cells CD45RA^–^CCR7^–^ effector memory T cells CD45RA^+^CCR7^–^ terminally differentiated effector T cells CD45RA^–^CCR7^+^ central memory T cells Activated CD4^+^ and CD8^+^ T cells = CD69^+^CD137^+^ Cytometric bead assay: IFNγ, TNFα, IL-2, IL-5, IL-13, IL-9, IL-10, and IL-22
Grifoni et al.^77^	Convalescent	Blood flow cytometry	OX40^+^CD137^+^CD4^+^ T cells (IL-2 and IFNγ) CD69^+^CD137^+^CD8^+^ T cells (IFNγ, TNFα, and granzyme B)
Zhou et al.^78^	Acute	Blood flow cytometry	CD69^+^CD38^+^CD44^+^OX40^+^CD4^+^ T cells (IL-6, GM-CSF, and IFNγ) CD69^+^CD38^+^CD44^+^CD137^+^CD8^+^ T cells (GM-CSF) PD-1^+^Tim-3^+^CD4^+^ T cells PD-1^+^Tim-3^+^CD8^+^ T cells CD16^+^CD14^+^ monocytes (IL-6 and GM-CSF)
Diao et al.^79^	Acute	Blood flow cytometry	PD-1^+^Tim-3^+^CD4^+^ T cells PD-1^+^Tim-3^+^CD8^+^ T cells Laboratory findings on cytokine levels: TNFα, IL-6, and IL-10
Zheng et al.^80^	Acute	Blood flow cytometry	NKG2A^+^CD8^+^ T cells (reduced secretion of CD107a, IFNγ, IL-2, and granzyme B) NKG2A^+^ NK cells (reduced secretion of CD107a, IFNγ, IL-2, granzyme B, and TNFα)
Ouyang et al.^81^	Acute	Blood CyTOF	CD4^+^ T cells* CD8^+^ T cells*
Leng et al.^83^	Acute	Blood CyTOF	CXCR3^+^CD4^+^ T cells CXCR^+^CD8^+^ T cells CXCR3^+^ NK cells
Wang et al.^82^	Acute	Blood CyTOF	CD4^+^CD8^+^ T cells Naïve CD4^+^ T cells TGFβ^+^CD28^–^ naïve CD4^+^ T cells Dendritic cells, macrophages, CD4^+^ T cells, and TGFβ^+^CD28^–^ naïve CD8^+^ T cells*^* Myeloid-derived suppressor cells (TGFβ, IL-10)
Liao et al.^85^	Acute	Lung BALF scRNAseq	CCR7^+^ central memory T cells ZNF683^+^CD8^+^ T cells FCN1^hi^ macrophages (*S100A8, FCN*, and *CD14*) FCN1^lo^SPP1^+^ macrophages (*CCL2, CCL3*, and *CXCL10*) SPP1^+^ macrophages (*A2M, GPR183*, and *CCL13*) FABP4^+^ macrophages (*FABP4, APOC1*, and *MARCO*) Upregulated expression of *IL1B, IL6, TNF, CCL2, CCL3, CCL4, CCL7, CXCL9, CXCL10, CXCL11, GZMA, GZMK*, and *FASLG* Cytometric bead assay: IL-6, IL-8, and IL-1β
**Wen et al.** ^87^	Convalescent	Blood scRNAseq	CD3E^+^CD4^+^ naïve T cells (*CCR7, LEF1*, and *TCF7*) CCR7^+^CD3E^+^CD4^+^ central memory T cells (*AQP3* and *CD69*) CD3E^+^CD4^+^ effector memory T cells (*CCR6, CXCR6, CCL5*, and *PRDM1*) FOXP3^+^CD3E^+^CD4^+^ Treg cells High expression of *FOS, JUN, KLF6, S100A8*, and *IL1B* CD8^+^ naïve T cells (*CCR7, LEF1*, and *TCF7*) CD8^+^ T_c_ cells (*GZMB, GNLY*, and *PRF1*) CD8^+^ effector memory T cells (*GZMK*)* TYMS^+^MKI67^+^CD8^+^ proliferating T cells *M-CSF, GM-CSF, IFNg*, and *IL-1β* Naïve B cells (*CD19, CD20, IL4R, TCL1A, IGHD*, and *IGHM*) Memory B cells (*CD27, CD38*, and *IGHG*) Immature B cells (*CD19* and *CD20*) Plasma cells (*XBP1* and *MZB1*) IL-6, *LTA*, and *LTB* IGHV3–7, *IGHV3–15, IGHV3–21, IGHV3–23*, and *IGHV3–30* *IGKV1–17, IGKV2–28*, and *IGKV3–15* *IGLV1–44, IGLV2–8*, and *IGLV3–27* CD14^+^IL-1β^+^ monocytes (*IL1B, JUN, FOS, JUNB*, and *KLF6*) CD56^hi^CD16^lo^ NK cells* CD56^lo^CD16^hi^ NK cells*

a The asterisk (*) indicates lower compared with healthy controls. The caret (^) indicates lower compared with non-ICU/nonsevere patients. hi, high; lo, low.

Flow cytometry and scRNAseq are also emerging tools. While they have limited applications in the field of diagnostics, they will be exceptionally valuable in research as the ability to conduct immunophenotyping allows for the identification of SARS-CoV-2-specific immune responses. By these approaches, we can elucidate the immune environment in patients with different severities of the disease and guide efforts to develop vaccinations against COVID-19.

**Figure 5.**
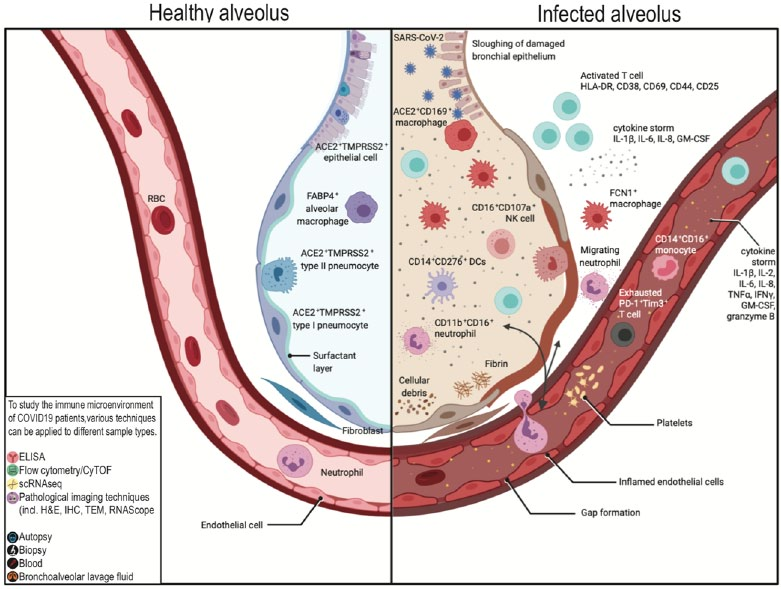
The alveolar immune microenvironment of patients with severe COVID-19 infection—comparison between healthy alveolus (left) and infected alveolus (right). As part of the SARS-CoV-2 antiviral response, pulmonary recruitment involves immune cells such as, but not limited to, (i) activated T cells, identifiable based on the expression of HLA-DR, CD38, CD69, CD44, and CD25; (ii) CD16^+^CD107a^+^ NK cells; (iii) CD11b^+^CD16^+^ neutrophils; (iv) FCN1^+^ macrophages; and (v) CD14^+^CD276^+^ dendritic cells. Recruitment of these pro-inflammatory immune cells results in a cytokine storm within the lung, as reported by elevated levels of IL-1β, IL-6, IL-8, and GM-CSF. This overall hyperinflammatory environment, when fueled by dysregulation of macrophage and lymphocyte populations in the lung, is a contributing factor to the observed lung function failure. In the blood immune microenvironment, despite consistent reports of lymphopenia, higher populations of CD14^+^CD16^+^ monocytes were observed. This is accompanied by a cytokine storm involving IL-1β, IL-2, IL-6, IL-8, TNFα, IFNγ, GM-CSF, and granzyme B, as well as an increase in functionally exhausted PD-1^+^Tim3^+^ T cells.

**Figure 6.**
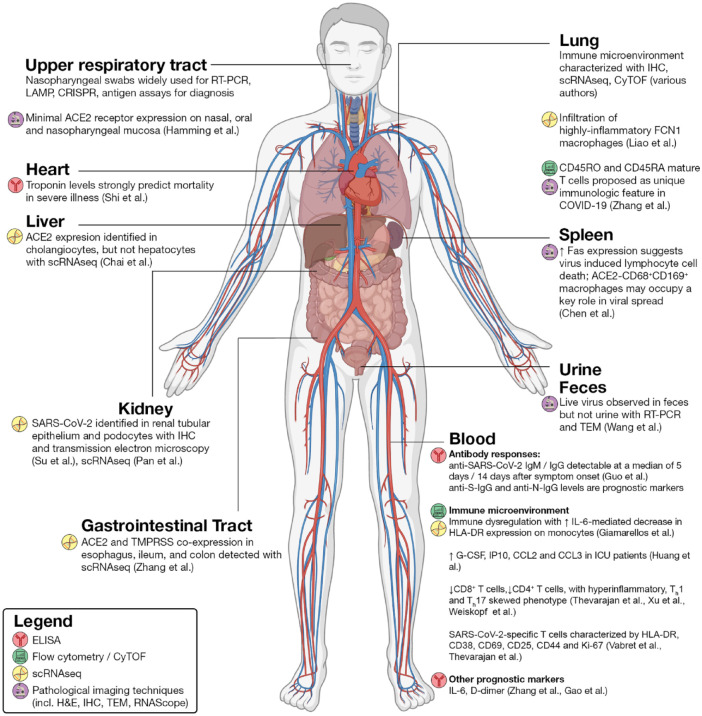
Selected findings by organ system. In the liver, scRNAseq has identified ACE2 expression predominantly on cholangiocytes, and cholangiocyte dysfunction has been speculated to explain liver injury. In the kidney, evidence of SARS-CoV-2 within renal tubular epithelium and podocytes suggests acute kidney injury as a primary element of severe COVID-19 infection. Within the gastrointestinal tract, ACE2 expression as well as detectable live virus in fecal samples indicates that fecal–oral transmission should be considered a possible route of transmission. In the lung, studies have characterized the immune microenvironment using pathological imaging approaches as well as scRNAseq and CyTOF. CD45RO and CD45RA mature T cells have been proposed as a unique immunologic feature in COVID-19. In the spleen and lymph nodes, ACE2^–^CD68^+^CD169^+^ macrophages are postulated to mediate SARS-CoV-2 translocation. CyTOF and scRNAseq approaches have also elucidated the extensive immune dysregulation at the heart of COVID-19. Immunological techniques like ELISA have also identified prognostic markers in serum such as troponin, IL-6, and D-dimer.

We have summarized the conclusions made by various authors regarding the immune microenvironment in the lung alveolus in **Figure 5** and in various organ systems in **Figure 6**. Although it is tempting to assume that these different techniques would generate correlating readouts, any conclusions should be interpreted with caution as each technique has its own limitations. For instance, side-by-side comparisons of ELISA, ELISPOT, and flow cytometry have shown discordant results in IFNγ secretion and expression levels.^70^ Single-cell analysis with flow cytometry compared against cyTOF or scRNAseq has also reported discrepancies in lymphocyte frequencies.^105^ Thus, we propose that these techniques are used in combination to provide more insights on the physiology of host immunity to COVID-19. Hopefully, these insights will be translated into clinical practice for disease management via the application of techniques like ELISA immunoassays to quantify important prognostic markers.

### Imaging Research Techniques

The importance of pathological imaging (through visual examination of injuries to organs, tissues, and cells) in augmenting our knowledge of the disease processes in COVID-19 cannot be ignored. Research surrounding COVID-19 imaging techniques predominantly involves the study of postmortem autopsy specimens of major organs such as the lungs, heart, liver, and kidneys. Approaches include conventional H&E staining, IHC staining, TEM, and emergent techniques such as the RNAscope. Immune cell types and viral interactions are recurring themes in most efforts, often identified through IHC staining of biomarkers such as CD4, CD8, CD20, and CD68, as well as ACE2, Fas, and SARS-CoV-2 S1 and N proteins.

#### Conventional Techniques

Conventional H&E and IHC staining are widely used approaches used to visualize alterations in tissue structure and the immune microenvironment in COVID-19. Lung pathology and the study of immune infiltrates is of particular interest, and reports from postmortem autopsies have consistently shown diffuse alveolar damage as the predominant finding within the lungs.^106,107^ Yao et al. performed H&E staining of pulmonary tissue from minimally invasive autopsies of three COVID-19 patients in China and identified varying degrees of alveolar destruction.^108^ IHC staining also identified CD68^+^ macrophage infiltration in the alveolar septum and cavity, and a small number of CD4^+^ T cells in the alveolar septum and interstitial lung, with the absence of CD8^+^ T cells and CD20^+^ B cells.^108^ Multiplex imaging approaches permit the simultaneous detection of a large number of markers; this technique is also starting to be adopted for complex analysis of tissues from COVID-19 patients. For instance, Zhang et al. examined lung tissue from COVID-19 patients with acute respiratory distress syndrome (ARDS) using a multiplex CyTOF imaging protocol that enabled the characterization of the precise identities and localization of mononuclear cell infiltrates.^109^ Interestingly, clusters of aberrant T cells, including CD45RO and CD45RA mature T cells and macrophages, which are typically more consistent with bacterial pneumonia, were also observed in these samples, and this was proposed to be a unique immunologic feature in COVID-19.^109^ Additionally, it should be noted that the methodology employed for viral detection in pathological samples for IHC varies; techniques range from staining for the viral NP antigen and viral S antigen to RT-PCR to visualization of the virus with TEM. An overview of the studies utilizing IHC staining and the biomarkers used is provided in **Table 4**.

**Table 4.** Studies Utilizing IHC and the Biomarkers Used.

Authors	Country	***N***	Study Design or Aims	Tissues	Techniques Used	IHC Markers Used	Viral Detection Method in Tissues
**Yao et al.** ^108^	China	3	Pathological report of three COVID-19 cases by minimally invasive autopsies	Lungs, heart, kidney, spleen, bone marrow, liver, pancreas, stomach, intestine, thyroid, skin	H&E, IHC, TEM	CK, CD68, CD20, CD8, CD4, TTF1,	IHC staining for SARS-CoV-2 spike S1, SARS-CoV NP, RT-PCR, TEM
**Su et al.** ^90^	China	26	Renal histopathological analysis of 26 postmortem findings of patients with COVID-19 in China	Kidneys	H&E, IHC, TEM	CD235a, CD61, CD31, ACE2, IgG, IgM,	IHC staining for SARS-CoV NP, TEM
**Luo et al.** ^121^	China	1	Performed whole-lung biopsy and described the pathological changes of critical COVID-19 patient done with transplant by H&E staining, immunohistochemistry, and special staining observed under the microscope	Lungs	H&E, IHC	CD3, CD4, CD5, CD8, CD20, CD38, CD68, CD79a, CK7, collagen IV	—
**Zhang et al.** ^109^	China	2	Described the type of immune cells identified by imaging CyTOF in lung tissue from two patients with COVID-19 and fatal ARDS	Lungs	H&E, IHC	CD4, CD68, CD16, CD107a, CD11b, CD16, CD45RA, CD45RO, CD4, CD276, CD14	—
**Chen et al.** ^97^	China	6	Through careful inspection of the spleens and lymph nodes from six cases with postmortem examinations, observed that SARS-CoV-2 could directly infect secondary lymphoid organs to induce cell death	Spleens, lymph nodes	H&E, IHC, TEM	ACE2, CD68, CD169, B220, Fas, FasL, IL-6	IHC staining for SARS-CoV-2 NP, TEM
**Xu et al.** ^106^	China	1	Investigated the pathological characteristics of a patient who died from severe infection with SARS-CoV-2 by postmortem biopsies	Lungs, liver, heart	H&E	—	—
**Tian et al.** ^122^	China	2	Two patients who recently underwent lung lobectomies for adenocarcinoma were retrospectively found to have had COVID-19 at the time of the operation	Lungs	H&E	—	—
**Barton et al.** ^107^	USA	2	Shared observations on the pathology of COVID-19 based on complete autopsies in two individuals who died in Oklahoma during the COVID-19 pandemic and were found to be positive for SARS-CoV-2 by postmortem testing	Lungs, heart, liver	H&E, IHC	CD3, CD4, CD8, CD20	RT-PCR
**Yan et al.** ^123^	USA	1	Presented a clinical-pathological correlation report of a previously healthy Hispanic woman with laboratory-confirmed COVID-19 who had typical features of ARDS and also showed cardiac abnormalities thought to represent fulminant viral myocarditis	Lungs, heart, kidneys	H&E, TEM	—	TEM
**Buja et al.** ^124^	USA	3	Collated the pathological findings from initial published autopsy reports on 23 patients with COVID-19 from 5 centers in the USA, including three cases from Houston, Texas	Lungs, heart, spleen, liver, kidneys, testis	H&E, IHC, TEM	CD3, CD4, CD8, CD61, CD68, TTF-1, CK-7, P40, CK5/6,	TEM
**Fox et al.** ^125^	USA	4	Reported on the cardiopulmonary findings of the first four autopsies of a series of 12 performed on patients within the USA, with relevant implications for the treatment of severe cases	Lungs, heart	H&E, IHC, RNA detection	CD4, CD8, CD61, VWF	RNA detection (DRAQ5 and SYTO RNASelect fluorescent staining)
**Bradley et al.** ^126^	USA	12	Addressed shortcomings by documenting a series of 12 fatal COVID-19 cases that occurred in Washington State during February–March 2020	Lungs, liver, brain, spleen, kidneys, large intestine	H&E, TEM, RT-PCR	—	RT-PCR
**Grimes et al.** ^127^	USA	2	Reported the finding of pulmonary thromboembolism as the cause of death in the initial two COVID-19 patients examined postmortem at their institution	Lungs	H&E, TEM	—	TEM
**Carsana et al.** ^128^	Italy	38	Systematically analyzed lung tissue samples from 38 patients who died from COVID-19 in two hospitals in northern Italy between February 29, and March 24, 2020	Lungs	H&E, IHC, TEM	CD45, CD68, CD61, TTF1, p40, Ki67, Masson Trichome	TEM
**Wichmann et al.** ^129^	Germany	12	Case series consisting of 12 consecutive autopsies	Heart, lungs, liver, kidneys, spleen, pancreas, brain, prostate, testes (in males), ovaries (in females), small bowel, saphenous vein, common carotid artery, pharynx, muscle	H&E, RT-PCR	—	RT-PCR
**Edler et al.** ^130^	Germany	80	Provided a systematic overview of the first 80 consecutive full autopsies	Heart, kidneys, liver, spleen, veins of lower extremities	H&E, RT-PCR	—	RT-PCR
**Ackermann et al.** ^131^	Germany	7	Examined seven lungs obtained during autopsy from patients who died from COVID-19 and compared them with seven lungs obtained during autopsy from patients who died from ARDS secondary to influenza A (H1N1) infection and 10 age-matched, uninfected control lungs	Lungs	H&E, IHC, TEM, micro-CT imaging, corrosion casting, direct multiplexed measurement of gene expression	CD3, CD4, CD8, CD15, CD20, CD61, CD68, podoplanin, ACE2, TMPRSS2, fibrinogen	TEM
**Varga et al.** ^132^	Switzerland	3	Demonstrated endothelial cell involvement across vascular beds of different organs in a series of patients with COVID-19	Lungs, kidneys, small bowel	H&E, IHC	Caspase 3	TEM
**Menter et al.** ^133^	Switzerland	21	Reported autopsy findings of 21 COVID-19 patients hospitalized at the University Hospital Basel and at the Cantonal Hospital Baselland, Switzerland	Lungs, liver, heart, brain, spleen, kidneys, pancreas, small intestine, large intestine	H&E, IHC, TEM	Fibrin, transthyretin (ATTR), CD3, CD4, CD8, CD20, CD68, MUM1, TTF1	RT-PCR
**Adachi et al.** ^134^	Japan	1	Reported an autopsy of an 84-year-old cruise ship passenger who died from COVID-19	All except the brain and bone marrow (lungs, spleen, lymph node, kidneys, stomach, small intestine, large intestine)	H&E, IHC	CD68	IHC staining for SARS-CoV NP RT-PCR

As mentioned, SARS-CoV-2 uses the ACE2 receptor for cellular entry. IHC staining for this biomarker can thus be informative in mapping its distribution and relationship with disease processes within the body in COVID-19 patients. For example, Su et al. found direct evidence of SARS-CoV-2 invading the renal tubular epithelium and podocytes, which are sites of known ACE2 expression;^90^ these findings provide support for acute kidney injury as a primary element of severe COVID-19 infection. Other authors have noted that within the liver, ACE2 receptors are localized to cholangiocytes but not hepatocytes;^110^ cholangiocyte dysfunction has thus been speculated to explain liver injury in COVID-19.^111^ Notably, one study reported that while IHC staining for ACE2 expression was negative in hepatocytes, a low frequency of ACE2 expression was identified by scRNAseq.^110^ This suggests that studies using IHC alone might be limited in their ability to accurately detect ACE2 expression. In sum, examinations of ACE2 cellular receptor expression via imaging research techniques are one essential avenue of research in the context of COVID-19 pathology.

Chen et al. performed IHC staining of spleen and lymph node tissue and found that Fas expression was significantly upregulated in infected patients but absent in sections from normal healthy controls.^97^ The researchers theorized that SARS-CoV-2-induced constitutional lymphocyte activation might trigger activation-induced cell death through Fas/FasL signaling via macrophagic IL-6 secretion. Besides lymphocyte migration into the lungs, this mechanism of viral killing of lymphocytes could be another component of the disease processes underlying the lymphopenia seen in COVID-19 patients.^97^ Additionally, IHC staining demonstrated ACE2 antigen present on CD68^+^CD169^+^ tissue-resident macrophages in the splenic marginal zone and lymph node marginal sinuses, together with evidence of SARS-CoV-2 invasion of these macrophages. The ACE2-expressing CD68^+^CD169^+^ macrophages were postulated to mediate SARS-CoV-2 translocation in spleens and lymph nodes, contributing to viral growth and spread. These intriguing findings on the interaction between SARS-CoV-2 and immune cells underscore our still limited understanding of the pathophysiology of COVID-19. Follow-up discussions on the possible implications in terms of disease management are now needed. Some caution, however, is urged as this study used the viral N antigen to identify SARS-CoV-2 virions, which have notable cross-reactivity with other coronaviruses.

#### Transmission Electron Microscopy

Several groups have used TEM as an approach to analyze parenchymal damage and to visualize viral particles and their distribution intracellularly within COVID-19 pathological samples. As mentioned, Su et al. used TEM to identify the presence of viral particles in the cytoplasm of the renal proximal tubular epithelium and podocytes, but with minimal quantities in the distal tubules.^90^ Similarly, Yao et al. also reported the presence of viral particles in the ciliated columnar epithelial cells of the bronchiolar mucosa.^108^

#### RNAscope

RNAscope technology is a novel emerging in situ hybridization (ISH) technique able to identify target RNA in cells using proprietary probes that simultaneously amplify target signal sequences while suppressing background noise from nonspecific hybridization.^112^ This technique can be used to visualize SARS-CoV-2 viral RNA and viral replication while precisely identifying vulnerable cell types by visualizing the cellular receptor ACE2 and proteases such as TMPRSS2, which facilitate viral entry into the host cells. For instance, Larsen et al. used RNAscope in a COVID-19-positive patient with collapsing glomerulopathy and described an absence of the SARS-CoV-2 virus in the kidney biopsy.^113^ These findings contrast with those from other authors derived from traditional IHC staining, where SARS-CoV-2 NP antigen was visualized in the renal tubules. When Larsen et al. attempted to replicate these findings with SARS-CoV-2 NP IHC staining of kidney tissues under numerous conditions, nonspecific positive staining in the renal parenchyma was observed.^113^ Alternatively, these contrasting findings might reflect a low sensitivity of RNAscope, producing false-negative results from viral antigens that are below the level of detection. This finding has substantial implications regarding the robustness of viral detection in other studies using IHC staining alone, and it would be prudent to conduct further evaluation of the concordance between conventional IHC methods and RNAscope technology.

ISH techniques such as RNAscope can also be multiplexed with IHC to visualize RNA and protein simultaneously.^114^ One report by Liu et al. discussed the development of a dual staining assay using IHC and RNAscope to detect SARS-CoV-2 antigen and RNA in the same formalin-fixed, paraffin-embedded section. The researchers demonstrated good consistency between the dual staining, as shown by the SARS-CoV-2 antigen being detected along with positive-sense RNA in the cytoplasm of most of the infected cells, but not in uninfected cells.^115^ This approach is promising for enabling highly precise detection of SARS-CoV-2, as positive IHC or ISH signals alone might merely indicate the presence of remaining free viral antigens or degenerating RNA fragments rather than actual viral particles.

#### Summary

In short, pathological imaging represents a rich source of knowledge in decoding the pathophysiology of COVID-19. We summarize the findings from studies utilizing pathological imaging, together with those from nonimaging approaches as discussed previously, in **Figures 5 and 6**. Our cumulative understanding of the mechanisms underlying this disease is likely to bear fruit in clinical practice in guiding our management of this pandemic. As with all pathological research, tissue availability remains a critical challenge for researchers. Further work is warranted to explore strategies to procure more biopsy or postmortem samples and to maximize yields from each tissue sample. Moreover, our opinion is that combining different techniques in multiplexed approaches offers one of the most compelling avenues for future research.

## Conclusion

In this review, we have discussed the major conclusions from virological, immunological, and imaging research approaches adopted to tackle the COVID-19 pandemic. We have also highlighted the key opportunities for future research surrounding COVID-19. Thus far, these three approaches have been instrumental in producing a robust, preliminary understanding of the disease (**Fig. 6**).

The battle against COVID-19 is far from over, but novel tools and scientific approaches are well placed at the forefront of the fight. Methods such as RNAscope and scRNAseq seem to be valuable supplements to current strategies, but further research is needed to evaluate their efficacy. For example, RNAscope might be even more specific than conventional IHC staining,^113^ and its combination with conventional IHC staining may allow the strengths of each technique to complement each other.^114^ scRNAseq and cyTOF approaches have also revealed fascinating insights about the immune microenvironment in patients with severe COVID-19. However, most work has focused on studying T cells in isolation. We believe that the detailed examination of multiple immune cell lineages (including dendritic cells, monocytes, and B cells) in parallel, using combinations of approaches, will potentially yield novel perspectives of the immune environment, such as a synergistic relationship between lymphocytes and myeloid cells in disease exacerbation. The increasing adoption of such multiplexed techniques bodes well for future research into this disease. Given the importance of research in COVID-19, many dedicated international consortiums, such as the COVID-19 High Performance Computing Consortium and the Crick COVID19 Consortium, have been established to coordinate research efforts in fighting COVID-19.

A critical concern is the persistence of SARS-CoV-2 in recovered patients. Discharged patients with COVID-19 seem to test positive for SARS-CoV-2 again.^116^ On a postmortem examination of a ready-for-discharge COVID-19 patient, SARS-CoV-2-viruses were found remaining in pneumocytes and there were also virus-induced pathological changes in the lungs.^19^ This bears chilling similarities to SARS-CoV, the closely related virus that caused the SARS pandemic in 2003. In a 6-year follow-up of confirmed SARS patients, it was found that the memory B-cell response to SARS-CoV was poor,^117^ which sets it apart from many other viral infections in which robust memory B-cell responses are elicited to produce neutralizing antibodies against future reinfections.^118^ Instead, the memory T-cell response was stronger in SARS-CoV patients;^117^ however, this response is not necessarily protective.^119^ Therefore, one question we must answer is whether COVID-19 shares the same susceptibility to reinfection as SARS. Given the important clinical and epidemiological implications posed by this question, it is imperative that future research is directed toward establishing the immunological response to SARS-CoV-2.

With immunological approaches being of great importance in the global effort against COVID-19, it raises another question: Is our trust in immunological approaches against COVID-19 misplaced? For example, Emirates airline recently became the first airline to implement rapid serology tests on passengers at the airport prior to boarding flights.^120^ However, as with all tests, rapid serological tests have their shortcomings and an accompanying host of problems. First, serological tests will not detect infections in its early stages when a humoral response has not yet been mounted. As such, a negative serological test may give false assurance to a person who may actually have been recently infected with SARS-CoV-2 and is still within the immunodiagnostic window period in which seroconversion has not yet occurred. Second, on-site rapid tests introduce a lot of unpredictability, as one will only know the results of the test at the site itself—this has many economic implications, for example, in terms of flight cancellations and insurance.

In conclusion, virological, immunological, and imaging research approaches have played a crucial role in the fight against COVID-19 and will surely continue to occupy center stage in the future. As the pandemic evolves, we hope that with the concerted efforts of researchers across the world, the pace of coronavirus research will outpace the spread of the virus. The ultimate aim is that our research efforts inform the development of vaccines and therapies for the disease, such that this pandemic can reach its resolution.
